# Deep-Learning-Based Segmentation of Extraocular Muscles from Magnetic Resonance Images

**DOI:** 10.3390/bioengineering10060699

**Published:** 2023-06-08

**Authors:** Amad Qureshi, Seongjin Lim, Soh Youn Suh, Bassam Mutawak, Parag V. Chitnis, Joseph L. Demer, Qi Wei

**Affiliations:** 1 Department of Bioengineering, George Mason University, Fairfax, VA 22030, USA; aquresh@gmu.edu (A.Q.);; 2 Department of Ophthalmology, Neurology and Bioengineering, Jules Stein Eye Institute, University of California, Los Angeles, CA 90095, USA; seongjinlim@mednet.ucla.edu (S.L.);

**Keywords:** deep learning, extraocular muscle, segmentation, MRI, strabismus, ophthalmology

## Abstract

In this study, we investigated the performance of four deep learning frameworks of U-Net, U-NeXt, DeepLabV3+, and ConResNet in multi-class pixel-based segmentation of the extraocular muscles (EOMs) from coronal MRI. Performances of the four models were evaluated and compared with the standard F-measure-based metrics of intersection over union (IoU) and Dice, where the U-Net achieved the highest overall IoU and Dice scores of 0.77 and 0.85, respectively. Centroid distance offset between identified and ground truth EOM centroids was measured where U-Net and DeepLabV3+ achieved low offsets (*p* > 0.05) of 0.33 mm and 0.35 mm, respectively. Our results also demonstrated that segmentation accuracy varies in spatially different image planes. This study systematically compared factors that impact the variability of segmentation and morphometric accuracy of the deep learning models when applied to segmenting EOMs from MRI.

## 1. Introduction

The visual and oculomotor systems comprise complex sensorimotor networks that must function properly to provide eye movement and alignment. Eye movements are executed by the six extraocular muscles (EOMs) and their pulleys. The motor commands to the EOMs are provided by the third, fourth, and sixth cranial nerves [1]. Abnormalities of the EOMs and cranial nerves can lead to strabismus. Strabismus, or “squint”, is binocular misalignment and is prevalent in 0.5–5% of the global population [2,3].

Imaging has played an important role in the examination of anatomical and biomechanical factors of strabismus as well as clinical management. Magnetic resonance imaging (MRI) was crucial in establishing the modern concept of pulley connective tissues of the extraocular rectus muscles (EOMs) [4]. In the past two decades, MRI-based anatomical and morphometric analysis has provided valuable knowledge of the neuro-biomechanics of eye movement for better management of strabismus [5,6,7,8].

Image-based examination of EOMs and other ocular structures have been performed through qualitative visual inspection of images by clinicians or quantitative morphometric analysis after these structures have been manually outlined in each image by trained experts. Manual segmentation is a time-consuming and labor-intensive process. For instance, in Hamwood (2021), the segmentation of the bony orbit can take about 4 to 8 h, depending on the complexity of the case for the patient [9].

To ease the labor burden of manual segmentation, researchers have developed approaches to semi-automate or automate the segmentation of ocular structures from medical images. Firbank (2000) implemented a semi-automated thresholding technique based on the edge of the muscles determined for each study subject’s MRI (19 euthyroid; 7 with thyroid-associated ophthalmopathy). They were only able to obtain approximately 25% of the EOM outlines by the semi-automated thresholding segmentation technique, where the rest had to be outlined manually [10]. Comerci (2013) implemented a relaxometric/geometric semi-automatic approach for measuring regional orbital fat in Graves’ ophthalmopathy in MRI. These authors first used manually segmented regions of the four rectus EOMs, fat, and other tissues and then automated the procedure for calculating the intra-orbital fat tissue volumes. Results showed low inter-operator variation (<5%) and high correlation with clinical data (*p* < 0.001) [11]. Xing (2015) utilized an automated segmentation method based on superpixels, region adjacency graphing, and normalized cuts while integrating prior shape information to learn and build upon the local information of the EOM regions rather than focusing on just pixels [12]. Segmentation results from 40 MRI images were assessed by computing the Rand index and the segmentation covering, which were 0.82 and 0.78, respectively, demonstrating the effectiveness of the proposed method.

Recently, automated ocular structure segmentation has shifted its focus of methodology to deep learning. Deep learning (DL) has recently become a fast-growing field of machine learning that seeks to model abstraction from large-scale data by employing multi-layered deep learning neural networks, thus making sense of data, such as MRI of the eye [13]. Deep learning-based techniques for image segmentation are based on semantic pixel-wise labeling [14]. Once the pixels are labeled, information can be gained based on the location and shape of the pixel-based regions. The models are expressed as a pixel classification problem with semantic labels, known as semantic segmentation [14,15]. These models include fully convolutional neural networks (FCN) such as the DeepLab series, U-Net architecture (FCN applied to biomedical images based on encoder–decoder architecture), Mask R-CNN, and others [15,16,17].

Zhu et al. (2021) implemented a 3D variant of the U-Net called the V-Net to segment four EOMs and the optic nerve from CT images [18]. Their V-Net model used 7413 images as training and 736 images as testing and achieved an overall intersection over union (IoU) of 0.8207. Shanker (2022) implemented U-Net on 178 training and 42 testing CT images [19]. The model outputted a Dice coefficient, a metric measuring similarity of two objects defined in Equation (2), of 0.92. Hamwood (2021) developed a 3-step deep learning method using the U-Net architecture for the segmentation of bony orbit on both MRI (366 training and 340 test) and CT scans (443 training and 363 test) [9]. The method achieved high Dice scores of 0.813 in CT and 0.93 in the MRI orbit segmentation.

Previous work on using DL in ocular structure segmentation showed promising results in terms of overlap between segmented and actual structure regions, as described above. However, we recognize two issues with existing analyses. First, obtaining segmented regions is not the ultimate outcome for many studies but facilitates the estimation of morphometric parameters such as EOM paths in three dimensions (3D). Therefore, an averaged measure of the region overlap may be inadequate in directly assessing the accuracy of using these DL models in morphometric analysis. Second, aggregated assessment metrics do not reveal what factors contribute to segmentation errors and thus cannot provide useful information to improve segmentation accuracy. A stack of 2D images obtained as contiguous cross-sections of 3D anatomical structures may have spatial characteristics and image acquisition settings that vary from slice to slice. For instance, when MRI data were obtained in our study, image contrast inevitably decreased for image slices progressively more remote from the surface coil near the cornea, making segmentation of remote slices more difficult. Other factors affecting segmentation include variable contrast of EOMs with a variety of surrounding tissues. These error sources are not captured by the conventional segmentation metrics such as the Dice coefficient or intersection over union score due to averaging.

The goal of our study was to compare the relative merits of existing DL segmentation models commonly used in medical image segmentation and apply them in the segmentation of EOMs in MRI. Our innovative thoughts are not only to evaluate the performance of the DL methods using the standard F-measure-based metrics commonly reported in the literature (IoU and Dice) in EOM segmentation from MRI but to assess suitability for estimating EOM morphometric characteristics such as centroid locations, which are crucial for 3D biomechanical modeling and clinical management of strabismus. Furthermore, we provide insights into the spatial factors affecting DL model performance, which can guide improvements in segmentation accuracy and contribute to the development of more robust and accurate DL models for ocular structure segmentation. Studies pertaining to the automated segmentation of ocular muscles from MRI images have been limited. Therefore, the insights gained from our study bring valuable guidance and inspiration to future researchers.

Our contributions to the field are threefold. First, we systematically compared four existing DL models on the same set of ocular MRIs, which had been carefully traced by an expert ophthalmologist to provide optimal image labels to train the DL models. Second, we addressed each model’s performance in determining the EOM centroid location in each image slice to evaluate morphometric performance. Third, to clarify any slice spatial factors affecting DL model morphometric performance, we systematically analyzed segmentation as a function of the MRI slice location.

The remainder of this paper is organized as follows. Section 2 describes the dataset and the methodology. Section 3 presents the results and the evaluation of the results, followed by a discussion in Section 4. Section 5 concludes the paper with remarks on the implications for future research.

## 2. Materials and Methods

### 2.1. Dataset

We studied MRI data collected for research purposes at UCLA, with prior written informed consent from each volunteer subject under a protocol approved by the Institutional Review Board of the University of California, Los Angeles. The images were obtained using a GE Medical System Signa 1.5T MRI scanner in quasi-coronal, 2 mm thick planes with 312 μm in-plane resolution using surface coils in a target-controlled central gaze.

The dataset contains orbital MRI stacks from 38 subjects (2 eyes per each subject; a total of 76 eyes). Of those subjects, 18 were normal, and 20 were diagnosed with superior oblique muscle palsy. Each MRI stack of one eye contained around 20 images with a 256 × 256 matrix. Both T1 and T2 MR images were included in the analysis. Image slices per eye that contained EOMs were extracted for pre-processing—of which the total number of usable MRI images in the dataset from the 76 eyes (38 subjects) resulted in 988. A few representative images of the dataset is uploaded in the GitHub page noted in the Supplementary Materials section.

### 2.2. Pre-Processing

**Figure 1:**
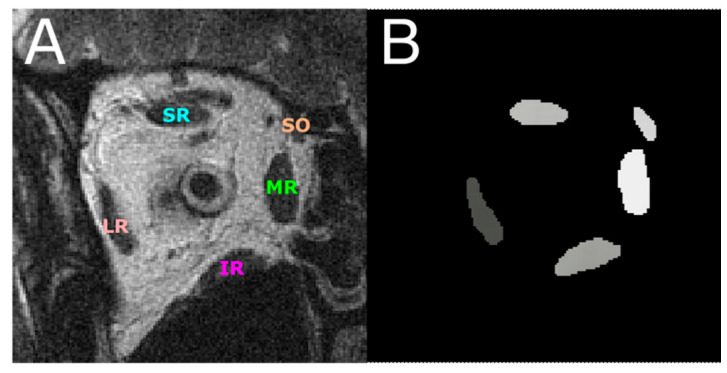
(**A**) An MR image with the five EOMs annotated. (**B**) EOMs were digitally traced from (**A**) and represented by grayscale regions of different pixel intensities. This image is used as the ground truth for training and testing DL segmentation models. LR—lateral rectus; MR—medial rectus; SR—superior rectus; IR—inferior rectus; SO—superior oblique.

In the 988 total TIF format images, the lateral rectus (LR), medial rectus (MR), superior rectus (SR), inferior rectus (IR), and superior oblique (SO) EOMs were digitally traced by hand using Fiji software to obtain a pixel-based mask in the region of interest (ROI) format to be used for the DL models as ground truth for pixel-based segmentation (Figure 1). The ROI files containing the ground truth traces were first converted in MATLAB in RGB color format to distinguish by color the five EOMs, making the segmentation a multiclass pixel-based segmentation problem. Each EOM region was then represented by a different pixel intensity, which formed a grayscale image mask for deep learning models (Figure 1B).

Sixty-four orbits were randomly chosen as training data, which consisted of 837 MR images in total and comprised about 85% of all traced MRI data. The remaining 12 orbits containing 151 MRI images were used in testing the trained DL models. No stratification was performed when training and test data were generated because our data was essentially balanced. Note that MRI stacks acquired from each orbit may contain varying numbers of image slices. Table 1 summarizes the training and testing data distribution.

### 2.3. Deep-Learning-Based Image Segmentation

Four deep learning models were implemented to solve this multiclass pixel-based segmentation problem: U-Net, U-NeXt, DeepLabV3+, and ConResNet. U-Net is a fully convolutional neural network (FCN) proposed by Ronneberger (2015) that is applied to biomedical image segmentation. It is based on the encoder–decoder architecture where the encoder module (contracting path captures context) and the expanding decoder module (expanding path) enable precise localization [16]. U-Net was evaluated by Yin (2022) as it is the methodology commonly used for biomedical image segmentation. Its advantages include fast-training speed and its suitability to be used on small datasets [20]. An extension of the U-Net is the U-NeXt [21]. The network combined a convolutional neural network and multilayer perceptron to reduce the computational cost. The DeepLab series (V1 to V3+) is a type of fully convolutional neural network that has been promising in determining the segmentation boundaries and has made use of conditional random fields (CRFs), which are used for structured prediction [22]. They have been used to segment regions of interest, such as the segmentation of the ciliary muscles [22]. In the current study, attention DeepLabV3+ was used for the comparison [23]. ConResNet is a 3D convolutional neural network developed by Zhang (2021) that uses the inter-slice difference between adjacent slices for 3D image segmentation [24].

### 2.4. DL Model Configuration

In our study, TensorFlow was used to implement U-Net, while U-NeXt, DeepLabV3+, and ConResNet were implemented using PyTorch. The models were tested on a computer with an AMD Ryzen 5 2600X six-core 3.60 GHz GPU and NVIDIA Quadro P6000 GPU. The models are uploaded in the GitHub page, listed in the Supplementary Materials section. Table 2 shows the model architecture parameters—including the number of epochs, the activation function, the loss function, the optimizer, and the number of trainable parameters. The number of epochs for training for each model was chosen to avoid overfitting. The number was determined empirically for each model through trial and error so that the difference between training accuracy and test accuracy was small.

### 2.5. Evaluation Metrics Methods

Upon running the models and obtaining the predicted masks, the four models were evaluated for their segmentation and morphometric measurement accuracies. To assess the segmentation effectiveness, we adopted two commonly used F-measure-based metrics, including the intersection over union (IoU) and the Dice coefficient [25]. These scores, in the range from 0 to 1, are generated by computing the relative overlap between the predicted EOM regions and the manually traced EOM regions. A score of 0 means that there is no overlap between the predicted EOM regions and the target regions, whereas a score of 1 means that the prediction perfectly matches the targets.

The definitions of the IoU and Dice metrics are shown in Equations (1) and (2), respectively,

(1) $$\mathrm{IoU}=\frac{TP}{TP+FP+FN}$$

(2) $$\mathrm{Dice}=\frac{2\times TP}{\left(TP+FP\right)+\left(TP+FN\right)}$$

where *TP* denotes true positive and includes those predicted pixels that were indeed part of the ground truth segmentation. False positive (*FP*) refers to those predicted pixels that were not in the target. False negative (*FN*) includes target pixels that were missed by DL segmentation.

Both IoU and Dice scores are reported in this manuscript for the sake of a comprehensive evaluation. In the three most relevant papers previously published on segmenting ocular structures, some used IoU for assessment [18], while others used Dice [9,19]. To achieve a fair comparison to prior work, we decided to compute both metrics.

The EOM path is an important morphometric parameter associated with strabismus. Certain types of strabismus, such as sagging eye syndrome (SES), are associated with EOM path elongation, which changes EOM function. Correlation of EOM paths from MRI with measured binocular misalignment can provide useful and quantitative knowledge of anatomical factors in strabismus. EOM paths can be approximated by curves connecting the centroids of EOM cross-sections in contiguous coronal images. Therefore, the accuracy of automatically determined EOM centroids is an indicator of the effectiveness of DL methods in generating EOM path estimates.

The predicted centroid error was calculated for each individual EOM in every image slice. The calculation of the Euclidean distance error between the manually segmented muscle centroid $\left(x_{1,\;}\;y_{1}\right)$ and the ground truth $\left(x_{2,}\;y_{2}\right)$ is given in Equation (3):

(3) $$\mathrm{Error}=\sqrt{{\left(x_{2}-x_{1}\right)}^{2}+{\left(y_{2}-y_{1}\right)}^{2}}$$

## 3. Results

### 3.1. Assessment of Segmentation Accuracy

**Figure 2:**
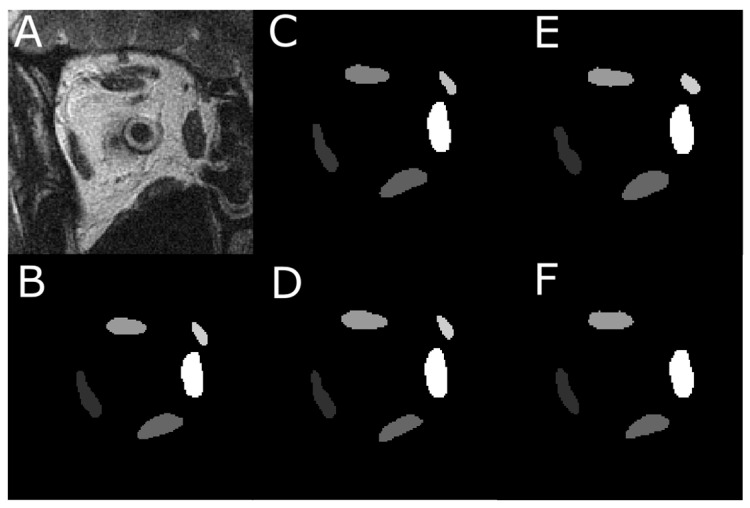
An instance of the multi-class pixel-based semantic segmentation where four DL models predicted the EOM regions. (**A**) Raw MRI image of the orbit. (**B**) Manually traced EOM regions in grayscale. Segmentation results from a trained (**C**) U-Net model, (**D**) U-NeXt model, (**E**) DeepLabV3+ model, and (**F**) ConResNet model.

**Figure A1:**
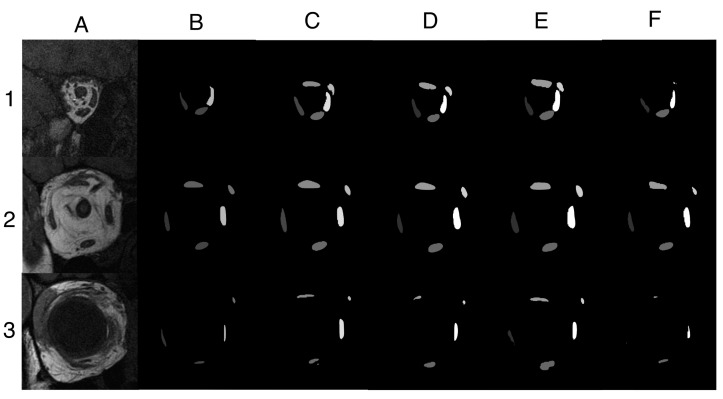
Three additional segmentation examples. (**A**) MRI images located (1) posteriorly, (2) near optic nerve–globe junction, and (3) anteriorly in the orbit. (**B**) Manually traced five EOM regions. Segmentation results from a trained (**C**) U-Net model, (**D**) U-NeXt model, (**E**) DeepLabV3+ model, and (**F**) ConResNet model. Example 1 demonstrated a scenario in which U-Net, U-NeXt, and DeepLabV3+ were able to segment the superior rectus muscle and superior oblique muscle accurately, which were missed by the operator. For Example 2, all four models were able to adequately segment the five EOMs, comparable to EOM regions traced manually. Example 3 was a challenging image in which the four rectus muscles were close to the globe and thus subject to poor contrast. Surprisingly, all four models were able to identify the superior rectus muscle, which was omitted by the operator. Lateral rectus muscle had low contrast to the surrounding tissue and was only segmented by DeepLabV3+. ConResNet was the least performed model for this case, while DeepLabV3+ seemed to be the best model overall. However, DeepLabV3+ over-segmented the inferior rectus muscle because it confused part of the inferior oblique muscle with the inferior rectus muscle because the two muscles were next to each other. In conclusion, variable segmentation efficacies were observed in different models and in different MRI images.

Each of the four models was first trained on 837 images with EOM masks and then tested on another 151 test images. Figure 1A shows one MRI slice in the test image set, from which EOM regions were segmented. Manually outlined EOMs were represented by areas of different grayscales shown in Figure 1B. Note that the mask in Figure 1B was not supplied to the DL models but was used to visually inspect DL results in Figure 2C–F, which contained EOMs automatically segmented by U-Net, U-NeXt, DeepLabV3+, and ConResNet, respectively. Through qualitative visual comparison, it can be concluded that these DL-generated EOM segmentations approximated EOM shapes and positions to the target regions manually identified by an ophthalmologist (Figure 1B). The only exception in this example image was that ConResNet failed to annotate the SO muscle (Figure 2F). Segmentation results of all 151 test images were visually examined. In 134 test images, EOMs were adequately identified without omitting the segmentation of one or more EOM or mislabeling the wrong EOM. Failure cases will be described in the Discussion section (Section 4). More example images can be found in Appendix A Figure A1.

**Figure 3:**
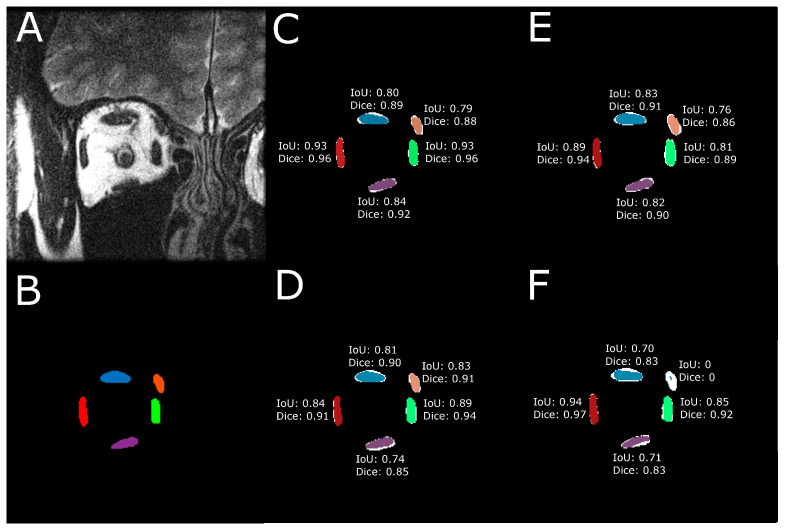
IoU and Dice scores were computed for every EOM. (**A**) Raw MRI image of the orbit. (**B**) Manually traced EOM regions. Segmentation results from a trained (**C**) U-Net model, (**D**) U-NeXt model, (**E**) DeepLabV3+ model, and (**F**) ConResNet model are shown as colored regions while the union of model segmentation and corresponding manual segmentation is visualized in white to show the discrepancies.

To quantitatively evaluate the performance of the model predictions against target EOM masks, IoU and Dice scores were computed for each of the five EOMs. Figure 3 illustrates the computed IoU and Dice scores using one example test image. In Figure 3B–F, the DL-based segmented EOMs are shown in color. The white region at each EOM is the union of the DL-segmented EOM region and the manually segmented EOM.

The means and standard deviations of IoU and Dice scored over all test images are reported in Table 3. The overall IoU and Dice scores averaged over all five EOMs were also computed. One-way ANOVA testing was performed on the IoU and Dice scores from the four models on the test dataset (151 images) and determined that the segmentation results were statistically significantly different among the four models (*p* < 0.001). Paired sample *t*-testing was also performed to compare the averaged performances of the four models.

**Figure 4:**
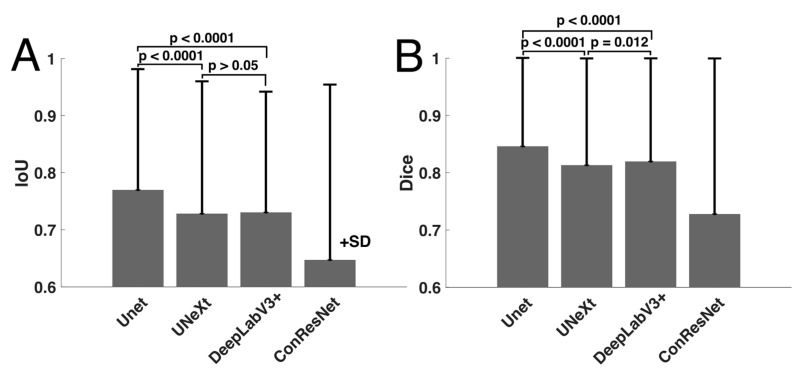
Comparison of mean (**A**) IoU and (**B**) Dice scores computed for the four models.

U-Net showed the highest averaged IoU score when all EOMs were considered. DeepLabV3+ and U-NeXt had similar IoU scores (*p* > 0.05) and were second best to U-Net (*p* < 0.001, Figure 4). ConResNet’s IoU was the lowest (*p* < 0.001). When individual EOMs were examined, U-Net again exhibited the highest mean IoU scores of all other models. IoU scores varied across different EOMs. The MR muscle had the highest IoU of all EOMs for every DL model. Furthermore, U-Net had the best MR segmentation, reaching a Dice score of 0.86. The SO muscle was the most difficult to segment. ConResNet only achieved an IoU score of 0.54 for SO. This was also the case for Dice scores, as summarized in Table 3 and Figure 4B, except that DeepLabV3+ has a slightly but significantly higher Dice score than U-Next (*p* = 0.012).

### 3.2. Assessment of EOM Centroid Estimation

**Figure 5:**
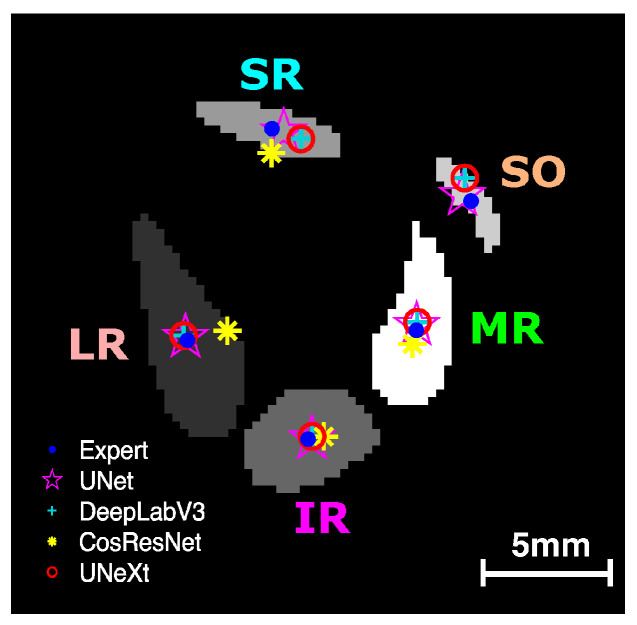
EOM centroids estimated from the segmented regions from manual segmentation and DL models. LR—lateral rectus; MR—medial rectus; SR—superior rectus; IR—inferior rectus; SO—superior oblique.

We analyzed the effectiveness of the four DL models in estimating EOM centroids from the segmented regions. Figure 5 shows an example of the centroid locations derived from the manual traces and the DL-generated segmentations. Centroids from U-Net, U-NeXt, and DeepLabV3 were all close to the centroids estimated from manual segmentation. The LR centroid resulting from ConResNet was clearly far from the target centroid, showing that EOM path estimates can vary with the different DL models used.

**Figure 6:**
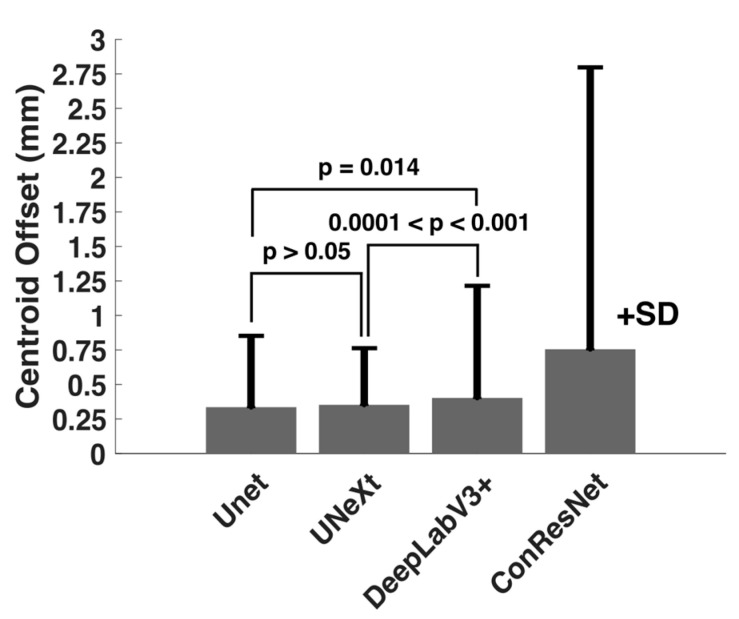
Comparison of mean centroid location offset (in mm) computed for the four models.

One-way ANOVA testing showed that the centroid distance offset in the test images for the four models varied significantly (*p* < 0.001). As Table 4 shows, U-Net had the least mean centroid error. However, the difference between the centroid errors of U-Net and DeepLabV3+ was not statistically significant (*p* > 0.05, Figure 6). Although DeepLabV3+ estimated segmented areas worse than U-Net, DeepLabV3+ was as effective as U-Net in estimating EOM centroid locations. Both U-NeXt and ConResNet were less effective (*p* ≤ 0.01, Figure 6). The centroid location offset of ConResNet averaged about 0.76 mm, which was more than twice the average U-Net centroid location error of about 0.34mm.

### 3.3. Cross-Validation

To evaluate the consistency of the DL model performance, we implemented cross-validation on the U-Net model, as it was regarded as the better-performing model. Five additional training–testing splits of 85–15% were produced, and corresponding testing accuracies of mean IoU, Dice, and centroid error are reported in Table 5. The observed accuracies show high consistency and generalization across different training–testing splits. This helps prove the validity of the DL models in the multiclass pixel-based segmentation problem.

### 3.4. Analysis of T-2 Weighted MRI Images

Our data consisted of a combination of 523 T1-weighted and 465 T2-weighted MRI images. One question worth studying is whether the observed performance accuracy would have been higher if a DL model was applied to images of the same MRI setting, a possible clinical setting.

A U-Net model using the same parameters in Table 2 was trained on 393 MRI images and tested on 72 images, both being T2-weighted. Table 6 summarizes the results of those 72 T2-weighted test images in terms of the averaged IoU, Dice, and centroid error. The averaged IoU score of U-Net from T2-weighted MRI images was 0.77 ± 0.20, and that from T1 and T2-weighted images was 0.77 ± 0.19. The averaged Dice scores from T2 MRI and T1 and T2 MRI were 0.85 ± 0.19 and 0.85 ± 0.18, respectively. The centroid offsets from T2 and combination were 0.31 and 0.34, respectively. An unpaired *t*-test (given average, standard deviation, and sample size) showed that the results were statistically insignificantly different for all categories (IoU *p*-value = 0.94; Dice = 1.0; centroid offset = 0.71) when comparing each metric in Table 6 against each metric in Table 3 and Table 4 for U-Net. Results suggest that the DL models evaluated in our study can handle mixed T1-weighted and T2-weighted MRI data and thus show good generality in realistic clinical settings.

### 3.5. Impact of MRI Slice Location on Segmentation Accuracy

**Figure 7:**
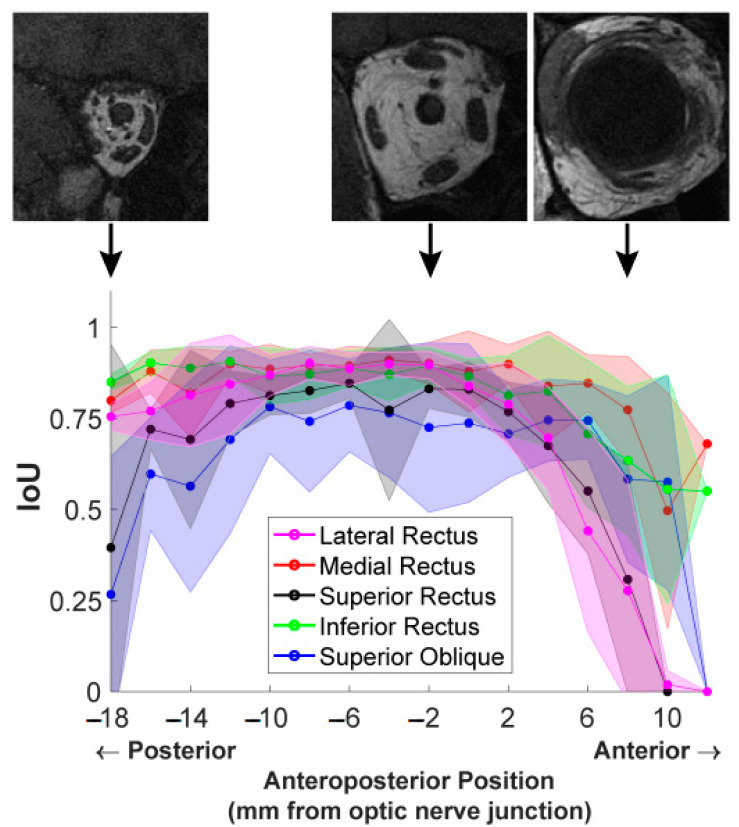
IoU scores from the U-Net model of EOMs averaged over all MR image slices at the same anteroposterior positions. Shaded areas show one standard deviation around the mean. Three representative T1-weighted MR images at anteroposterior positions of −18 mm, −2 mm, and 8 mm are placed displaced at corresponding positions on the graph to show the different anatomical characteristics.

Finally, we examined the influence of anteroposterior MR image slice location on segmentation accuracy. Figure 7 shows the computed IoU scores for the five EOMs at each image slice. To analyze segmentation results at corresponding locations across different orbits, all 2 mm thick MRI images in test data were spatially aligned anteroposteriorly, referring to the slice at the optic nerve–globe junction as location zero. For each EOM, the IoU scores of all test images at the same anteroposterior position were averaged, and the resultant mean values were plotted in Figure 7, providing a comparison of the performance of each DL model for each EOM according to the anteroposterior position.

The IoU score of each EOM varied with anteroposterior positions according to an inverted U-shaped curve. IoU score was much lower for the most anterior and posterior positions and was greatest in mid-orbit. The extent of the anteroposterior region of high IoU varied among EOMs. For instance, the IoU plotted for the MR in red was high over a longer range from the orbital apex (−18 mm) to 8 mm anterior to the globe–optic nerve junction, anterior to IoU significantly decreased. The IoU for the LR had a shorter anterior range, declining significantly by 2 mm posterior to the globe. Segmentation quality markedly dropped in slices more than 4 mm anterior to the globe–optic nerve junction, likely because EOMs transition to thin tendons in this region and are closely surrounded by dense connective tissue and other anatomical structures. For the LR and SR, IoU even dropped to zero at an anterior position of 12 mm, indicating DL models failed to find these EOMs at that position while the ophthalmologist expert was able to trace the EOMs. Different EOMs have individually varying IoU values as well as different patterns of IoU variation with the anteroposterior position. These results implied that it is insufficient to report only one overall IoU averaged over slices of different locations and over different EOMs, as typically used in reporting DL segmentation performance. Segmentation accuracy is specific both to the individual EOM and to the specific anteroposterior location.

**Figure 8:**
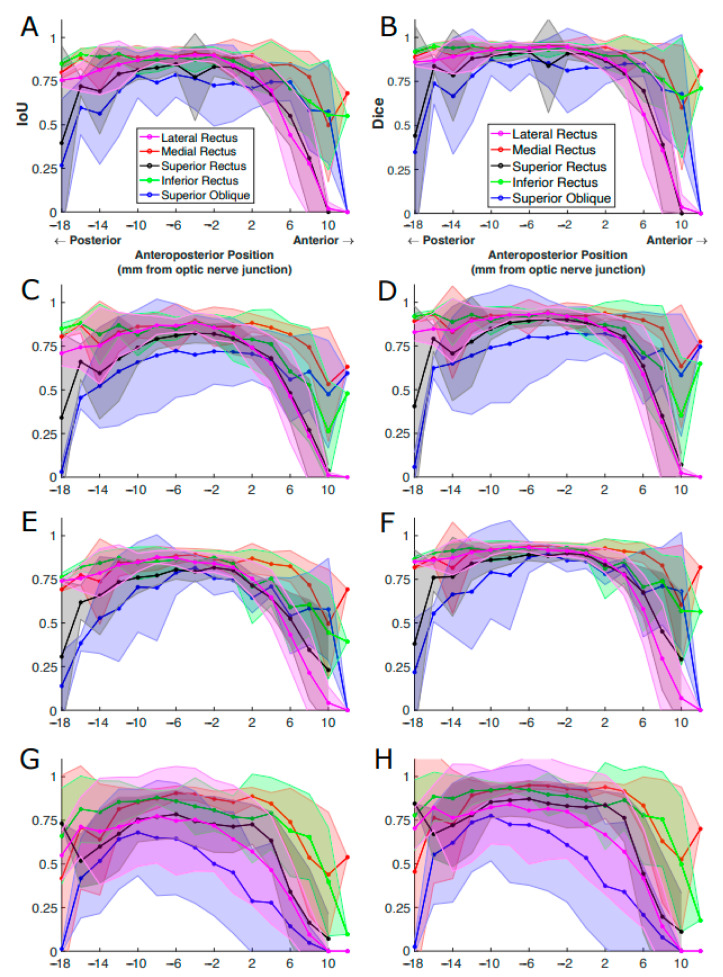
IoU and Dice scores of EOMs averaged over all MR image slices at the same anteroposterior positions. The results were from (**A**,**B**) U-Net, (**C**,**D**) U-NeXt, (**E**,**F**) DeepLabV3+, and (**G**,**H**) ConResNet. The shaded areas show the standard deviations associated with the mean values. (**A**) is the same as Figure 7 for ease of comparison.

We performed corresponding analysis on decomposed IoU and Dice scores computed for four DL models. A similar relationship between segmentation accuracy and slice position was observed (Figure 8). Although ConResNet had the worst overall performance, it was still able to segment MR and IR comparably to the other three models. ConResNet segmented the SO and LR poorly and inconsistently, as evident by the large standard deviations.

Accuracy varied with anteroposterior slice location due to both systematic variation in the EOM cross-section and EOM contrast and decreasing signal-to-noise ratio (SNR) with posterior distance in the orbit. The top three MR images in Figure 7 were from the same stack; however, the anatomical characteristics of EOMs, such as their sizes and contrasts, are significantly variable at different anatomical locations. Posterior image planes are difficult to segment because the EOM cross-sections are small and crowded against other structures, while the image SNR is lower due to a longer distance from the surface coil located near the face. More anteriorly in the mid-orbit, fat surrounding large, well-isolated EOM cross-sections and a higher SNR made labeling EOM regions easier. Anterior to the optic nerve–globe junction, EOMs become thinner, and highly contrasting fat surrounding them is replaced with dense connective tissues having MRI signals similar to EOMs. In the most anterior orbit, it becomes difficult or impossible to distinguish EOM tendons from the globe and other connective tissues. The use of gadodiamide intravenous contrast can further improve EOM contrast against the connective tissue of surrounding pulleys; however, contrast was not employed in this study [26].

### 3.6. Impact of MRI Slice Location on Estimated EOM Centroids

**Figure 9:**
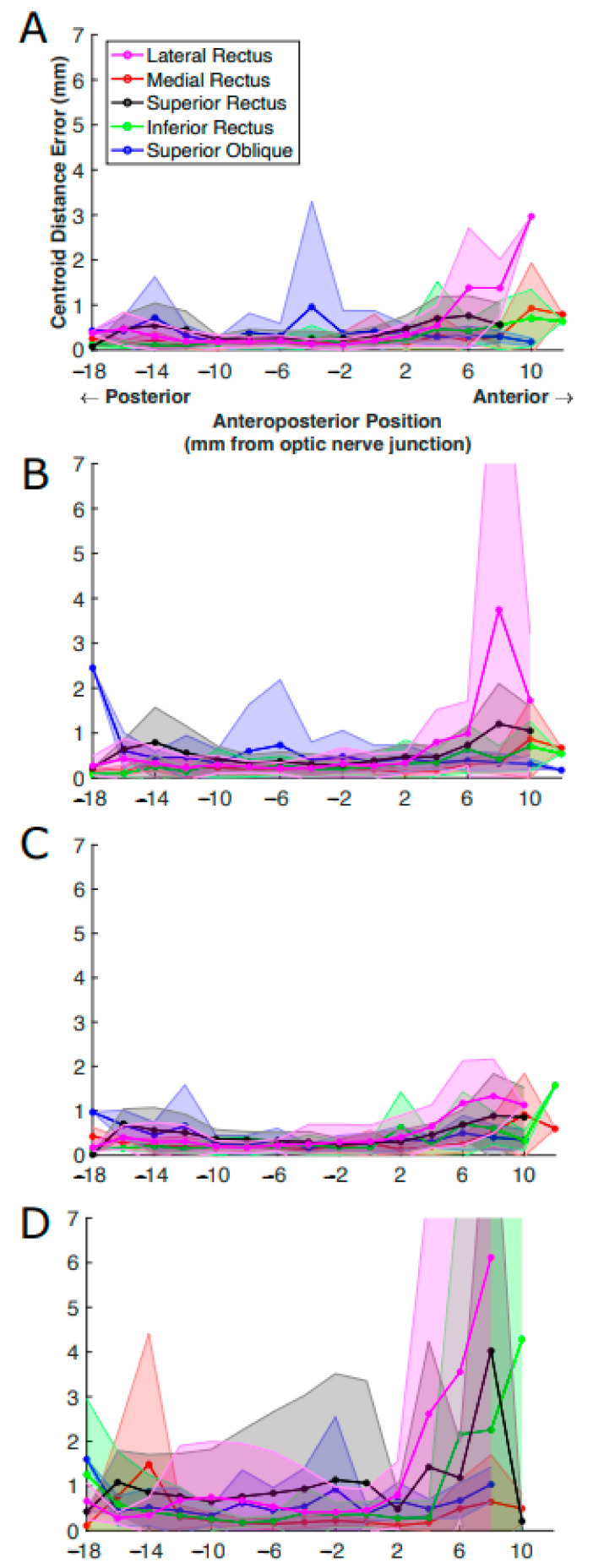
Centroid distance error (in mm) per EOM averaged over all MR image slices at the same anteroposterior positions. The results were from (**A**) U-Net, (**B**) U-NeXt, (**C**) DeepLabV3+, and (**D**) ConResNet. The shaded areas show the standard deviations associated with the mean values.

We examined the impact of slice position on estimated EOM centroid locations. As shown in Figure 9, imperfections in segmenting EOMs from anterior images were clear in all models, with ConResNet showing deviation of the centroid of the EOM from ground truth both posterior and anterior to the optic nerve junction point. When there is inaccuracy in the estimation of the centroid from the ground truth, this can lead to errors in the analysis of the EOM function and motion when translating the data into 3D biomechanical models. Based on Figure 9, possible errors in EOM function and motion analysis can arise when taking the anterior/insertion region of the EOM of the models into account as the regions are harder to segment, and the centroid is therefore challenging to determine relative to ground truth.

### 3.7. Computation Costs of DL Models

The computational expense of the four models was documented and compared (Table 7). ConResNet was the most computationally efficient model in terms of both training time per epoch and total training time but was associated with the worst segmentation performance. DeepLabV3+ took the longest computational time per epoch, twice as much as U-Net and U-NeXt. However, DeepLabV3+ was performed for only 25 epochs, making it the second most efficient model computationally. U-Net took the longest to train, doubling the time needed for U-NeXt.

## 4. Discussion

Traditionally, the analysis of the structures from biomedical images has been time and labor-intensive and is subject to operator errors. The advancement of ML techniques opens the opportunity to make medical image analysis automated and objective. In particular, the DL methods reduce the workload to segment large image sets with minimal post-processing by physicians and researchers [27].

Among the four DL models tested, U-Net, U-NeXt, and DeepLabV3+ all achieved a mean Dice score exceeding 0.81. Previous work on DL-based EOM segmentation was performed on CT images [18,19]. The best performed reported was a Dice score of 0.92 for the four rectus EOMs, benefiting from the injection of Omnipaque contrast dye during CT image acquisition [19]. The only other DL segmentation on the ocular MRI only reported results on the bony orbit with a Dice score of 0.93 [9]. Nevertheless, we identified a few important issues that need to be considered when ML-based segmentation is used in clinical image analysis. An in-depth interpretation of results derived from ML models is necessary for their usage in disease research in labs and diagnosis in clinics.

**Figure 10:**
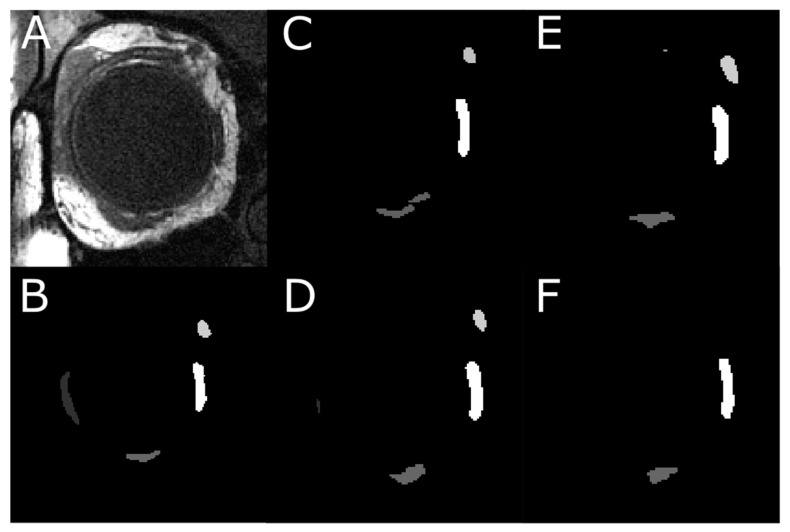
An instance of segmentation where the models do not perfectly segment the regions of interest. (**A**) An MR image located anteriorly in the orbit. (**B**) Digitally traced four EOMs. (**C**) Result from U-Net model, which identified SO and MR, did not manage to segment LR, and mislabeled the inferior oblique muscle as IR. (**D**) Result from U-NeXt model, which under-segmented LR. (**E**) Result from DeepLabV3+ model, which also missed LR. (**F**) Result from ConResNet model, which was only able to segment MR and IR.

Aggregated measurements such as IoU and Dice are commonly used to assess segmentation accuracy. The underlying assumption is that the variability among images and different structures is limited, so averaged performance suffices to report effectiveness. However, as shown in Figure 8, performance metrics vary appreciably with anatomical location. The peak accuracy is observed at the EOM muscle belly in mid-orbit and decreases closer to the EOM insertion and origin. An example of such a case is in Figure 10, located anterior to the optic nerve junction point where the performance of the DL models suffers due to the EOMs being obscured by the eyeball—making it harder for the expert ophthalmologist to trace them, let alone the DL models to predict them.

While our study has demonstrated the effectiveness of using DL methods for EOM segmentation on MRI, it is not without its limitations. This study did not investigate the impact of model hyperparameters on segmentation accuracy. The model performances are expected to improve with optimized model hyperparameters; however, they were not conducted due to limited time. We did not thoroughly examine the potential of data augmentation techniques to improve performance, especially in the posterior and anterior portions of the eye. Further studies are planned to examine the impact of the performance using data augmentation and model hyperparameter tuning. Finally, the eye socket bone, optic nerve, and eyeball were not segmented and should be included in future studies.

Usually, machine-learning-based automatic image segmentation benefits from having a large training dataset. However, it is also recognized that obtaining large and heterogeneous labeled medical images for training is a tedious task and can even be impossible for clinical data. In our study, it took quite some time to generate the 988 labeled images, in which five extraocular muscles were accurately digitally traced. Approaches have been proposed to address the issue of a lack of large training data. Transfer learning has been shown to be an effective strategy for image classification. Instead of training a model from scratch, transfer learning enables learning a new model that leverages patterns in a pre-trained model to solve a similar problem from a large dataset [28]. The effectiveness of improved MRI segmentation accuracies when using pre-trained models in similar domains has been discussed in many papers [29,30,31]. Data augmentation is another commonly used method to increase training medical image data size and thus allows deep learning models to be implemented with boosted variability of the data [32]. Zhang et al. (2020) reported that data augmentation methods could improve DL performances by 12–47% [33]. Fabian (2021) investigated augmentation using MRI datasets. Using pixel-preserving augmentations and general affine augmentations, improved performances were observed. They did note that the implementation of augmentation should carefully be considered as the incorrect implementation can significantly lower performance compared to not using augmentation [34]. In our future work, we plan to adopt transfer learning and data augmentation to improve the segmentation accuracy on unlabeled MRI images.

Other improvements would be to introduce weights on the different MRI slices based on their relative anatomical locations to strengthen the learning on the most anterior and posterior slices. By incorporating these improvements into the DL models, they can be better trained on the challenging regions of the eye. Ultimately, the development of 3D biomechanical models could be developed to investigate the anatomical relationships within MRI stacks of the eyes—allowing us to obtain a better understanding of the spatial data of EOM cross-sections in adjacent slices.

It is important to examine DL segmentation performance using non-area overlap metrics that are meaningful in anatomical and morphometric analysis. For many applications, defining the segmented region is not the ultimate goal. The primary goal is often to use quantified EOM paths to build subject-specific biomechanical orbit models to simulate the consequence of EOM lesions and to quantitatively compare the effectiveness of surgical procedures [35].

From a biomechanical modeling perspective, the accuracy of estimating EOM areas is as important as the accuracy of obtaining EOM paths through connecting the centroids of EOMs in MRI slices. Therefore, conclusions on the DL model effectiveness should not be based solely on area overlap assessment metrics but also on morphometric representation accuracy. As our results showed, although IoU and Dice scores for DeepLabV3+ were inferior to U-Net, DeepLabV3+ is as effective as U-Net in locating EOM centroids. Since DeepLabV3+ is computationally more efficient, it can be a good candidate for time-sensitive applications.

## 5. Conclusions

Our study has demonstrated that the DL models can generally accurately segment the five EOMs in the high-resolution, quasi-coronal MRI images of the human orbits based on both visual and quantitative assessment. Automated segmentation through DL can help reduce the time involved in manual segmentation while also reducing the potential for operator-dependent errors. We investigated the factors that can impact the segmentation accuracy and the EOM centroid offsets—primarily the location of the MRI slices relative to the optic nerve junction point, which can impact the subsequent analysis of EOM biomechanical function based on geometry and size of the EOMs.

We also pointed out the limitation of using IoU and Dice to assess segmentation performance when functional anatomy is to be studied. This study was not without its limitations, including the lack of model parameter optimization and the usage of data augmentation, which would be appropriate to be implemented in future studies, as this paper was to establish baselines on which model(s) would be best to use for EOM segmentation from MRI.

## Figures and Tables

**Table 1:** Training–testing split of the data representing the total number of eyes and images.

	Number of Eyes	Number of Images	Percentage
Training	64	837	85%
Testing	12	151	15%
Total	76	988	

**Table 2:** Architecture parameters of the four DL models tested.

	U-Net	U-NeXt	DeepLabV3+	ConResNet
Epochs	200	100	25	15
Activation Function	ReLU/Softmax	ReLU/GELU	ReLU	ReLU
Loss Function	Categorical Loss Entropy	Cross Entropy and Dice	Cross Entropy	Cross Entropy
Optimizer	Adam	Adam	Adam	Adam
Number of Trainable Parameters	1,940,902	1,471,989	65,197,632	22,169,272

**Table 3:** Segmentation performance of four DL models on 151 test images. IoU and Dice were computed for individual EOMs as well as their averages over all test images. SD—standard deviation.

		U-Net	U-NeXt	DeepLabV3+	ConResNet
**IoU** **(mean** ** ± SD)**	MR	0.86 ± 0.13	0.83 ± 0.14	0.82 ± 0.14	0.84 ± 0.09
	SO	0.70 ± 0.23	0.64 ± 0.25	0.65 ± 0.23	0.54 ± 0.27
	IR	0.83 ± 0.15	0.78 ± 0.21	0.78 ± 0.17	0.80 ± 0.16
	SR	0.71 ± 0.23	0.68 ± 0.23	0.68 ± 0.21	0.69 ± 0.16
	LR	0.75 ± 0.26	0.72 ± 0.26	0.72 ± 0.25	0.65 ± 0.29
	**Averaged**	**0.77 ± 0.20**	**0.73 ± 0.22**	**0.73 ± 0.21**	**0.70 ± 0.19**
**Dice** **(mean** ** ± SD)**	MR	0.92 ± 0.11	0.90 ± 0.13	0.89 ± 0.13	0.91 ± 0.06
	SO	0.79 ± 0.21	0.74 ± 0.26	0.76 ± 0.23	0.66 ± 0.27
	IR	0.90 ± 0.12	0.85 ± 0.20	0.87 ± 0.15	0.88 ± 0.14
	SR	0.80 ± 0.24	0.78 ± 0.23	0.78 ± 0.21	0.80 ± 0.14
	LR	0.82 ± 0.25	0.80 ± 0.26	0.80 ± 0.25	0.74 ± 0.27
	**Averaged**	**0.85 ± 0.19**	**0.81 ± 0.21**	**0.82 ± 0.21**	**0.80 ± 0.18**

**Table 4:** Predicted EOM centroid location error.

		U-Net	U-NeXt	DeepLabV3+	ConResNet
**Centroid Error (mm)**	MR	0.23 ± 0.33	0.25 ± 0.26	0.25 ± 0.29	0.37 ± 0.92
	SO	0.41 ± 0.77	0.46 ± 0.62	0.36 ± 0.36	0.56 ± 0.69
	IR	0.25 ± 0.37	0.29 ± 0.31	0.29 ± 0.38	0.72 ± 2.70
	SR	0.41 ± 0.35	0.50 ± 0.43	0.44 ± 0.39	1.02 ± 2.06
	LR	0.40 ± 0.59	0.53 ± 1.59	0.42 ± 0.54	1.09 ± 2.60
	**Averaged**	**0.34 ± 0.52**	**0.40 ± 0.81**	**0.35 ± 0.40**	**0.76 ± 2.03**

**Table 5:** Cross-validation of U-Net segmentation of EOMs. Five additional training–testing splits were generated and tested.

Average Performance U-Net (200 eps)						
	Split 1 (Reported in Table 3 and Table 4)	Split 2	Split 3	Split 4	Split 5	Split 6
**IoU** **(mean ± SD)**	0.77 ± 0.20	0.79 ± 0.18	0.78 ± 0.20	0.77 ± 0.21	0.77 ± 0.20	0.79 ± 0.19
**Dice** ** (mean ± SD)**	0.85 ± 0.19	0.87 ± 0.17	0.85 ± 0.18	0.87 ± 0.21	0.85 ± 0.19	0.86 ± 0.18
**Centroid Error** **(mean mm ± SD)**	0.34 ± 0.52	0.27 ± 0.34	0.31 ± 0.49	0.29 ± 0.35	0.30 ± 0.45	0.33 ± 0.76

**Table 6:** Average metrics for U-Net model tested on T2-weighted images. Each pixel for centroid error is equivalent to 312 microns.

	IoU (Mean ± SD)	Dice (Mean ± SD)	Centroid Error (mm) (Mean ± SD)
MR	0.85 ± 0.13	0.91 ± 0.12	0.18 ± 0.16
SO	0.66 ± 0.21	0.77 ± 0.21	0.38 ± 0.29
IR	0.79 ± 0.20	0.86 ± 0.20	0.28 ± 0.32
SR	0.74 ± 0.24	0.82 ± 0.24	0.36 ± 0.30
LR	0.80 ± 0.16	0.88 ± 0.14	0.35 ± 0.65
**Averaged**	**0.77 ± 0.19**	**0.85 ± 0.18**	**0.31 ± 0.34**

**Table 7:** Comparison of training time of the four DL models.

	U-Net	U-NeXt	DeepLabV3+	ConResNet
**Number of Epochs**	200	100	25	15
**Time for One Epoch (second)**	0.97	1.08	2.09	0.29
**Total Training Time (second)**	197	108	52.25	4.35

## Data Availability

Not applicable.

## Supplementary Materials

A GitHub repository that contains the trained DL models and a few representative MRI images has been created and can be assessed using the following link: https://github.com/AAQ2017/EyeProject.
